# Author Correction: Genotypic variability-based genome-wide association study identifies non-additive loci HLA-C and IL12B for psoriasis

**DOI:** 10.1038/s10038-019-0605-5

**Published:** 2019-05-23

**Authors:** Wen-Hua Wei, Jonathan Massey, Jane Worthington, Anne Barton, Richard B. Warren

**Affiliations:** 1 Arthritis Research UK Centre for Genetics and Genomics, School of Biological Sciences, Faculty of Biology, Medicine and Health, Manchester Academic Health Science Centre, University of Manchester, Oxford Road, Manchester, M13 9PT UK; 2 Department of Women’s and Children’s Health, Dunedin School of Medicine, University of Otago, Dunedin, 9016 New Zealand; 3 NIHR Manchester Musculoskeletal Biomedical Research Unit, Central Manchester NHS Foundation Trust, Manchester Academic Health Science Centre, Manchester, M13 9PT UK; 4 Dermatology Centre, Salford Royal NHS Foundation Trust, Manchester Academic Health Science Centre, University of Manchester, Manchester, M6 8HD UK

**Correction to:** **J Hum Genet**

https://www.nature.com/articles/s10038-017-0350-6

Published online 19 December 2017.

This article was originally published under a CC BY-NC-SA License, but has now been made available under a CC BY 4.0 License.

